# A Simple, Cost-Effective, and Robust Method for rRNA Depletion in RNA-Sequencing Studies

**DOI:** 10.1128/mBio.00010-20

**Published:** 2020-04-21

**Authors:** Peter H. Culviner, Chantal K. Guegler, Michael T. Laub

**Affiliations:** a Department of Biology, Massachusetts Institute of Technology, Cambridge, Massachusetts, USA; b Howard Hughes Medical Institute, Massachusetts Institute of Technology, Cambridge, Massachusetts, USA; University of Pittsburgh

**Keywords:** RNA sequencing, rRNA depletion, subtractive hybridization

## Abstract

The profiling of gene expression by RNA sequencing (RNA-seq) has enabled powerful studies of global transcriptional patterns in all organisms, including bacteria. Because the vast majority of RNA in bacteria is rRNA, it is standard practice to deplete the rRNA from a total RNA sample such that the reads in an RNA-seq experiment derive predominantly from mRNA. One of the most commonly used commercial kits for rRNA depletion, the Ribo-Zero kit from Illumina, was recently discontinued abruptly and for an extended period of time. Here, we report the development of a simple, cost-effective, and robust method for depleting rRNA that can be easily implemented by any lab or facility. We first developed an algorithm for designing biotinylated oligonucleotides that will hybridize tightly and specifically to the 23S, 16S, and 5S rRNAs from any species of interest. Precipitation of these oligonucleotides bound to rRNA by magnetic streptavidin-coated beads then depletes rRNA from a complex, total RNA sample such that ∼75 to 80% of reads in a typical RNA-seq experiment derive from mRNA. Importantly, we demonstrate a high correlation of RNA abundance or fold change measurements in RNA-seq experiments between our method and the Ribo-Zero kit. Complete details on the methodology are provided, including open-source software for designing oligonucleotides optimized for any bacterial species or community of interest.

## Abstract (precis)

The ability to examine global patterns of gene expression in microbes through RNA sequencing has fundamentally transformed microbiology. However, RNA-seq depends critically on the removal of rRNA from total RNA samples. Otherwise, rRNA would comprise upward of 90% of the reads in a typical RNA-seq experiment, limiting the reads coming from mRNA or requiring high total read depth. A commonly used kit for rRNA subtraction from Illumina was recently unavailable for an extended period of time, disrupting routine rRNA depletion. Here, we report the development of a “do-it-yourself” kit for rapid, cost-effective, and robust depletion of rRNA from total RNA. We present an algorithm for designing biotinylated oligonucleotides that will hybridize to the rRNAs from a target set of species. We then demonstrate that the designed oligonucleotides enable sufficient rRNA depletion to produce RNA-seq data with 75 to 80% of reads coming from mRNA. The methodology presented should enable RNA-seq studies on any species or metagenomic sample of interest.

## INTRODUCTION

RNA sequencing (RNA-seq) is a common and powerful approach for interrogating global patterns of gene expression in all organisms, including bacteria (1–3). In most RNA-seq studies, it is desirable to eliminate rRNAs so that as many reads as possible come from mRNAs (4). For most eukaryotes, the majority of mRNAs are polyadenylated, enabling their selective isolation and subsequent sequencing (5, 6). In contrast, bacteria do not typically polyadenylate their mRNAs, and rRNA comprises 80% or more of the total RNA harvested from a given sample (7). To enrich for mRNA in RNA-seq samples, a general strategy involves the depletion of rRNAs by subtractive hybridization (8–10). This approach was at the heart of commercially available kits such as Ribo-Zero from Illumina, leading to RNA-seq data in which ∼80 to 90% of the reads map to mRNAs. Despite the popularity and efficacy of Ribo-Zero, this kit was abruptly discontinued by the manufacturer for an extended period of time, which disrupted many RNA-seq users and pipelines that depend on it.

Here, we report an easily implemented, scalable, and broadly applicable do-it-yourself (DIY) rRNA depletion kit. Our kit relies on the physical depletion of rRNA from a complex RNA mixture using biotinylated oligonucleotides specific to 5S, 16S, and 23S rRNA. We focus primarily on the development of oligonucleotides that will enable depletion of rRNA from any one of eight different, commonly studied bacteria. However, we also present an algorithm for customizing the subtractive oligonucleotides, and the open-source software developed here can be used to design oligonucleotides for the depletion of rRNA from any user-defined set of species. Our results indicate that the kit we developed enables the facile depletion of rRNA from total RNA samples such that ∼70 to 80% of reads in RNA-seq map to mRNAs. We further demonstrate that our kit produces RNA-seq data showing high correspondence to that produced using the Ribo-Zero kit. Additionally, our kit has a reduced cost of only ∼$10 per sample to deplete rRNA from 1 μg of total RNA. We anticipate that this rRNA-depletion strategy will benefit the entire bacterial community by enabling low-cost transcriptomics with a similar workflow as the Ribo-Zero kit.

## RESULTS

**FIG 1:**
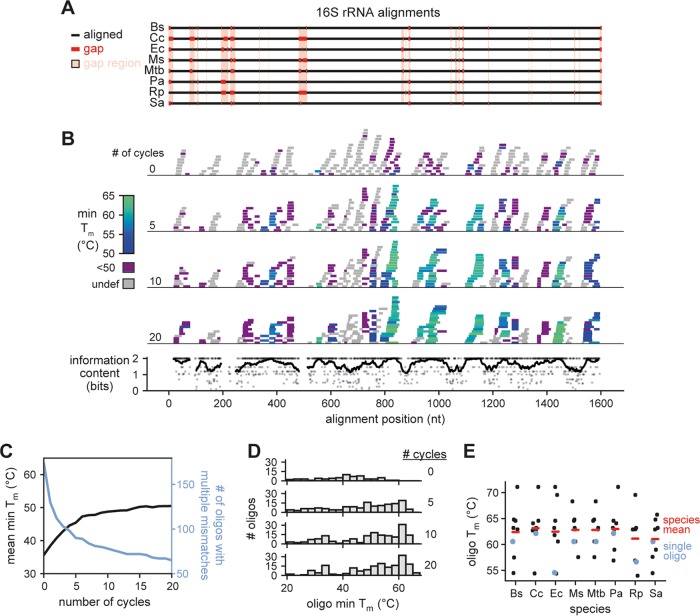
Oligonucleotide selection for 16S rRNA. (A) Alignment of 16S sequences from 8 bacterial species (Ec, E. coli; Pa, P. aeruginosa; Rp, *R. parkeri*; Cc, C. crescentus; Bs, B. subtilis; Ms, M. smegmatis; Mtb, M. tuberculosis; Sa, S. aureus). Alignment gaps are shown as red lines in the particular species of the gap. Regions with a gap in any species are highlighted in pink; these regions were not considered when designing oligonucleotides. (B) The position, length, and minimum *T_m_* of all oligonucleotides plotted against the 16S alignment after the indicated number of optimization cycles (top). The information content at each nucleotide position of aligned regions is also shown (bottom, points). To highlight conserved regions, a sliding average information content is also plotted (bottom, line). (C) Oligonucleotide *T_m_* statistics after multiple cycles of the *T_m_* optimization algorithm. For each oligonucleotide (*n* = 250), we calculated the minimum *T_m_* across the 8 species considered and then plotted the mean of this value across all oligonucleotides (black). The *T_m_* cannot be accurately estimated for oligonucleotides with multiple sequential mismatches; the number of oligonucleotides with an undefined *T_m_* is also plotted (blue). (D) Histograms of minimum *T_m_* for oligonucleotides at the indicated number of optimization cycles. Data were generated as in panel C, but oligonucleotide *T_m_* minima were used to generate histograms rather than taking the mean across all oligonucleotides. Oligonucleotides with an undefined *T_m_* were not included in the histograms. (E) Distribution of *T_m_* values for each 16S-targeting oligonucleotide (*n* = 8) for each individual species indicated. The mean *T_m_* of oligonucleotides for each species is also shown (red lines). Note that the same oligonucleotides are used for each species, but because of 16S sequence variability, the *T_m_* can vary, as illustrated for one particular oligonucleotide (blue).

To efficiently and inexpensively deplete rRNA from total RNA from multiple organisms, we developed an algorithm to design DNA oligonucleotides capable of hybridizing to rRNA from multiple species simultaneously. We reasoned that each rRNA should be bound by multiple oligonucleotides across the length of the rRNA, in case a given site is hidden by structure or is not available due to partial fragmentation during RNA extraction or processing. Further, we decided that oligonucleotides should be as short as possible to reduce synthesis cost and decrease the likelihood of spurious binding and accidental depletion of mRNA. To find potential binding sites, we aligned the 16S and 23S rRNA sequences from a set of eight bacteria (Escherichia coli, Pseudomonas aeruginosa, Rickettsia parkeri, Caulobacter crescentus, Bacillus subtilis, Mycobacterium smegmatis, Mycobacterium tuberculosis, and Staphylococcus aureus), including several major pathogens and model organisms (Fig. 1A; see also Fig. S1A in the supplemental material) (16S and 23S rRNA, respectively). These sequences were divergent enough that we could not design an oligonucleotide based on the rRNA sequence of a single species and expect it to bind the rRNA from other species effectively. Thus, we designed an algorithm to optimize the sequence of oligonucleotides, enabling them to hybridize to rRNA from multiple species. To find these oligonucleotides, we focused on ungapped regions of the alignment and randomly chose a large number of sites as candidates (Fig. 1B and Fig. S1B). For nucleotide positions that were completely conserved among the eight species, the conserved nucleotide was selected. For positions that were only partially conserved, a nucleotide was chosen at random such that it would match a nucleotide found in some but not all of the rRNAs.

10.1128/mBio.00010-20.1 FIG S1 Oligonucleotide selection for 23S rRNA. (A) Alignments of all 23S sequences from 8 bacterial species (Ec, E. coli; Pa, P. aeruginosa; Rp, *R. parkeri*; Cc, C. crescentus; Bs, B. subtilis; Ms, M. smegmatis; Mtb, M. tuberculosis; Sa, S. aureus). Alignment gaps are shown as red lines in the particular species of the gap. Regions with a gap in any species are highlighted in pink; these regions were not considered when designing oligonucleotides. (B) The position, length, and minimum *T_m_* of all oligonucleotides plotted against the 23S alignment after the indicated number of optimization cycles (top). The information content at each nucleotide position of aligned regions is also shown (bottom, points). To highlight conserved regions, a sliding average information content is also plotted (bottom, line). (C) Oligonucleotide *T_m_* statistics after multiple cycles of the *T_m_* optimization algorithm. For each oligonucleotide (*n* = 500), we calculated the minimum *T_m_* across the 8 species considered and then plotted the mean of this value across all oligonucleotides (black). The *T_m_* cannot be accurately estimated for oligonucleotides with multiple sequential mismatches; the number of oligonucleotides with an undefined *T_m_* is also plotted (blue). (D) Histograms of minimum *T_m_* for oligonucleotides at the indicated number of optimization cycles. Data were generated as in panel C, but oligonucleotide *T_m_* minima were used to generate histograms rather than taking the mean across all oligonucleotides. Oligonucleotides with undefined *T_m_* were not included in the histograms. (E) Distribution of *T_m_* values for each 23S-targeting oligonucleotide (*n* = 11) for each individual species indicated. The mean *T_m_* of oligonucleotides for each species is also shown (red lines). Note that the same oligonucleotides are used for each species, but because of 23S sequence variability, the *T_m_* can vary, as illustrated for one particular oligonucleotide (blue). Download FIG S1, TIF file, 1.9 MB. Copyright © 2020 Culviner et al. 2020 Culviner et al. This content is distributed under the terms of the Creative Commons Attribution 4.0 International license.

We then performed an iterative process to sample alternate sequences and binding locations for each oligonucleotide, while biasing the selection toward sequences that tightly bind rRNA from the eight species we had selected. To do this, for each oligonucleotide we generated *in silico* a set of mutated oligonucleotides that varied from the original sequence by either extending, shrinking, or shifting the binding site, or by mutating a single nucleotide of the oligonucleotide to match a different species’ rRNA. From this set of mutated oligonucleotides, the algorithm effectively replaced each old oligonucleotide with a new one, favoring those close to a target minimum melting temperature (*T_m_*) of 62.5°C. This *T_m_* was chosen to achieve tight binding across all species while preventing selection of excessively long oligonucleotides. Our chosen *T_m_* estimation algorithm did not allow multiple adjacent mismatches between the oligonucleotide and the RNA; these oligonucleotides we considered to have undefined *T_m_* values. Oligonucleotides with defined *T_m_* values were always favored during optimization. With more cycles of optimization, the average minimum *T_m_* approaches the target *T_m_* (Fig. 1C and Fig. S1C). Notably, many oligonucleotides, particularly those with poorly conserved start locations, were not able to reach the target *T_m_*, though the number that did increased with additional cycles (Fig. 1D and Fig. S1D). After 15 to 20 cycles, the oligonucleotides had converged on highly conserved regions of the rRNAs (Fig. 1B to D and Fig. S1B to D). After 100 cycles of optimization, we selected 8 and 9 nonoverlapping oligonucleotides for the 16S and 23S rRNA, respectively, with an average length of 30 nucleotides. These 17 oligonucleotides are predicted to hybridize to rRNA from all eight species included in the initial design. Although the *T_m_* for individual oligonucleotides varies across species, the mean *T_m_* for the oligonucleotide set as a whole was similar (Fig. 1E and Fig. S1E).

We also applied our algorithm to the 5S rRNA from the 8 species considered. However, because the 5S rRNA is both shorter and more poorly conserved than 16S and 23S rRNA, we were unable to find oligonucleotides that are predicted to effectively hybridize to the 5S rRNA from all eight species. Therefore, we ran the algorithm against individual 5S rRNAs and hand-selected two oligonucleotides specific to the 5S from each species. In addition, we found that the algorithm was unable to find oligonucleotides mapping near the 5′ and 3′ ends of the 23S due to its low conversation among species. To improve binding to these regions, we also identified two oligonucleotides that were specific to either Gram-positive or Gram-negative members of our target set of species. Thus, our final set of depletion oligonucleotides for a given organism includes 21 total oligonucleotides: 17 common oligonucleotides targeting 16S and 23S rRNA, 2 oligonucleotides that target 23S rRNA in a Gram-positive- or Gram-negative-specific manner, and 2 species-specific 5S targeting oligonucleotides (Table S1; oligonucleotides each contain a 5′-biotin modification).

10.1128/mBio.00010-20.4 TABLE S1 Sequences of oligonucleotides for bacterial rRNA depletion. Download Table S1, PDF file, 0.03 MB. Copyright © 2020 Culviner et al. 2020 Culviner et al. This content is distributed under the terms of the Creative Commons Attribution 4.0 International license.

**FIG 2:**
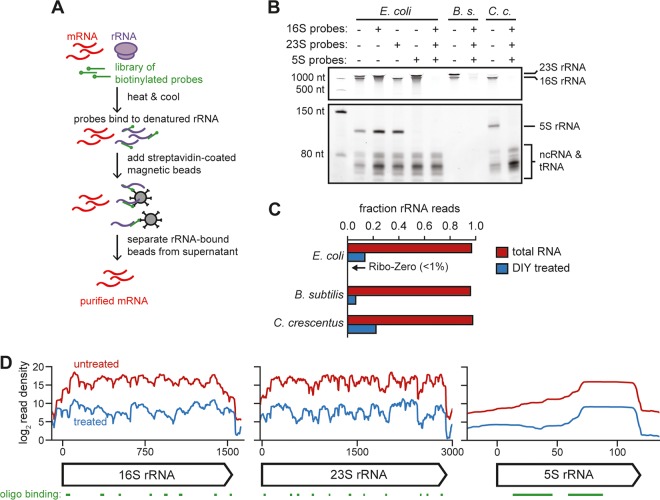
rRNA depletion by oligonucleotide-based hybridization. (A) Cartoon of the rRNA depletion process. (B) Polyacrylamide gel showing total RNA from E. coli, B. subtilis, and C. crescentus before and after rRNA depletion using indicated probe sets. The first lane is a ladder. Approximate positions of abundant RNAs, including rRNAs, are indicated on the right. Note that a lower contrast is shown for the top portion of the gel to resolve 16S and 23S bands. B. subtilis RNA extraction partially depleted the 5S and small ncRNAs (see Materials and Methods). (C) Fraction of total reads aligning to rRNA for rRNA-undepleted and -depleted samples of E. coli, B. subtilis, and C. crescentus total RNA. (D) Summed read counts across the E. coli 16S, 23S, and 5S rRNAs before (red) and after (blue) depletion. The positions of oligonucleotides used for depletion are shown below.

We then sought to determine whether our oligonucleotide libraries could effectively deplete bacterial rRNA. To deplete rRNAs from a total RNA sample, we incubated biotinylated versions of the 21 designed oligonucleotides with total RNA. Samples were then combined with magnetic streptavidin-coated beads to precipitate the oligonucleotides bound to rRNAs, followed by isolation of the supernatant, which should be heavily enriched for mRNA (Fig. 2A). We extracted total RNA from exponentially growing cultures of common lab strains of E. coli, B. subtilis, and C. crescentus and performed a single round of rRNA depletion. For all three species, incubation with the 21 depletion oligonucleotides substantially decreased the intensity of rRNA signal on a polyacrylamide gel, while tRNA and ncRNA were generally unaffected (Fig. 2B). Moreover, this depletion was modular, as incubation of E. coli total RNA with probes targeting only 16S, 23S, or 5S rRNA resulted in selective depletion of the band corresponding to a given targeted rRNA (Fig. 2B, left).

To quantify how well our method depleted rRNA, we performed RNA-seq before and after rRNA depletion for E. coli, B. subtilis, and C. crescentus. We then calculated the fraction of reads mapping to rRNA loci in each case (Fig. 2C). The fraction mapping to rRNA decreased following depletion from >95% to 13%, 6%, and 22%, respectively. Our method was also effective at depleting a 1:1:1 mixture of total RNA from these species (from 96% to 17%; Fig. S2A). Though this depletion is less efficient than Ribo-Zero, it is adequate for most sequencing studies. Improved depletion is likely possible with the addition of more depletion oligonucleotides and streptavidin beads, though this increases the price per sample (see Materials and Methods). To determine whether there was any bias for depletion of certain regions of the rRNAs, we compared read counts at each nucleotide position pre- and postdepletion in each E. coli rRNA (Fig. 2D). For the 16S, 23S, and 5S rRNAs, read density was relatively uniform but lower, following depletion, indicating that no particular region of the rRNAs (e.g., regions prone to high structure or partial degradation, preventing effective depletion) was overrepresented in our rRNA reads.

**FIG 3:**
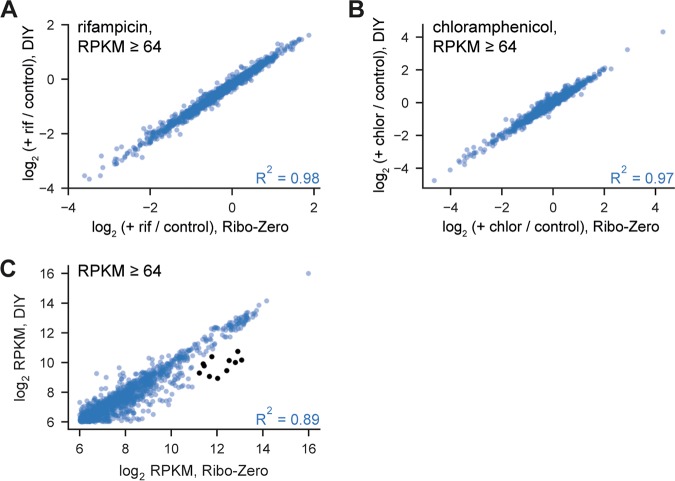
Our rRNA depletion strategy performs comparably to Ribo-Zero for RNA-seq. (A) Scatterplot showing correlation between log_2_ fold change for E. coli coding regions following rifampicin treatment, comparing rRNA depletion via Ribo-Zero with our depletion strategy. Fold changes were calculated as the ratio of RPKM between rifampicin-treated and untreated samples. All coding regions with at least 64 RPKM in both untreated samples (*n* = 1,294) were considered in the analysis. (B) Scatterplot showing correlation between log_2_ fold change for E. coli coding regions following chloramphenicol treatment, comparing rRNA depletion via Ribo-Zero with our depletion strategy. Fold changes were calculated as the ratio of RPKM between chloramphenicol-treated and untreated samples. All coding regions with at least 64 RPKM in both untreated samples (*n* = 1,294) were considered in the analysis. (C) Scatterplot showing correlation between read counts (RPKM) for E. coli coding regions treated with Ribo-Zero and our do-it-yourself (DIY) depletion strategy. All coding regions with at least 64 RPKM in both samples (*n* = 1,294) were considered in the analysis. Eleven outliers preferentially depleted by our method are highlighted (black); also see Fig. S2D.

10.1128/mBio.00010-20.2 FIG S2 Analysis of outliers in correlation between mRNA counts following Ribo-Zero and DIY rRNA depletion. (A) Fraction of total reads aligning to rRNA for rRNA-undepleted and -depleted samples for a 1:1:1 mixture of E. coli, B. subtilis, and C. crescentus total RNA. Fractions were calculated for all species in aggregate as well as for the individual species. (B) Scatterplots showing correlation between read counts (RPKM) for E. coli coding regions treated with Ribo-Zero and our do-it-yourself (DIY) depletion strategy for RNA extracted from cells grown in M9 (left) and LB (right). All coding regions with at least 1 count in both samples (*n* = 4,214 and 4,265) are shown. Outliers at least 2 standard deviations away from the least squares fit (*n* = 241 and 235) are shown. Those more depleted in our method are plotted in black, and those more depleted in Ribo-Zero are shown in gray. (C) Scatterplots showing correlation between log_2_ fold changes for E. coli coding regions following rifampicin (left) and chloramphenicol (right) treatment, comparing rRNA depletion via Ribo-Zero with our depletion strategy. Fold changes were calculated as the ratio of RPKM between treated and untreated samples. All coding regions with at least 1 count in all samples (*n* = 4,157 and 4,203) were considered in the analysis. Shared outliers (*n* = 163, Table S2) from Fig. S2B are plotted in black (more depleted in our method) and gray (more depleted in Ribo-Zero). (D) Figure 3A (left) and B (right), with outliers identified in Fig. 3C marked in black. Correlations are listed for all plotted genes (blue) and outliers (black). Download FIG S2, TIF file, 2.2 MB. Copyright © 2020 Culviner et al. 2020 Culviner et al. This content is distributed under the terms of the Creative Commons Attribution 4.0 International license.

Many RNA-seq studies are aimed at detecting significant differences in the expression of mRNA in different strains or across different perturbations. Treatment with antibiotics causes rapid, significant, and well-characterized changes to bacterial transcriptomes. Thus, to ensure that our depletion technique did not affect the measurement of expression changes (e.g., through unintended depletion of particular mRNAs), we treated E. coli cells with either rifampicin or chloramphenicol for 5 min and compared fold changes measured from libraries generated using our depletion strategy to those generated using the previously available commercial kit Ribo-Zero (Illumina). For each depletion method, we calculated the log_2_ fold change in read counts in coding regions following antibiotic treatment compared to a negative control (Fig. 3A and B). For both rifampicin and chloramphenicol treatment, the correlation in log_2_ fold change per highly expressed coding region between the two rRNA depletion strategies was high (*R*^2^ = 0.98 and 0.97 for rifampicin and chloramphenicol, respectively) across a wide range of changes in gene expression. These results indicate that our method should provide similar results as the Ribo-Zero kit for studies measuring changes in gene expression.

Importantly, we also determined if our kit differentially depleted particular mRNAs compared to the Ribo-Zero kit. To do this, we directly compared the RPKM (reads per kilobase per million) values for highly expressed coding regions from a library prepared using our depletion strategy and one prepared using Ribo-Zero (Fig. 3C). Overall, there was a high correlation between the two depletion methods among highly expressed genes (*R*^2^ = 0.89). However, there were a few apparent outliers that were more depleted by our method than by Ribo-Zero. To formally test if outliers drive technical error in the calculation of expression fold change during perturbations, we first plotted all coding regions for two independent comparisons of libraries made with our method and Ribo-Zero (Fig. S2B). In both comparisons, there was a high correlation between the two depletion methods, even for low-expressed genes (*R*^2^ = 0.95 and 0.94). For each comparison, we called genes more than 2 standard deviations away from a log-linear fit of RPKM as outliers. Of the 241 and 235 outliers in the two comparisons, 163 outliers were present in both. Next, to determine if these shared outliers drove error in fold change calculation, we compared the fold change values calculated for all E. coli coding regions in libraries prepared using our method and Ribo-Zero (Fig. S2C; *R*^2^ = 0.73 and 0.77 for rifampicin and chloramphenicol, respectively). Outliers more depleted by our method (black, *R*^2^ = 0.67 and 0.66) or by Ribo-Zero (gray, *R*^2^ = 0.90 and 0.66) did not show any apparent biases in fold change values (Fig. S2C). We also hand-selected 11 of the most highly expressed outliers that were more significantly depleted by our method than Ribo-Zero (Fig. 3C, black dots). Again, the fold changes in expression calculated following treatment with rifampicin or chloramphenicol were highly correlated for the two depletion methods (Fig. S2D; *R*^2^ = 0.96 and 0.89 for rifampicin and chloramphenicol, respectively). Thus, results obtained based on our oligonucleotide hybridization approach are highly comparable to those generated with the commercial Ribo-Zero kit.

Finally, we compared the RPKM values for highly expressed coding regions in libraries prepared using our depletion strategy and libraries from total RNA (no rRNA depletion) from E. coli, B. subtilis, and C. crescentus (Fig. S3A). Depleted libraries for each species showed similar correlations, with *R*^2^ = 0.75, 0.73, and 0.71 for E. coli, B. subtilis, and C. crescentus, respectively. In addition, libraries prepared from the same E. coli RNA samples with Ribo-Zero and our method showed comparable correlations to a library built from undepleted RNA (Fig. S3B; *R*^2^ = 0.60 and 0.71 for our method and Ribo-Zero, respectively). These results confirm that our method is an effective strategy for depleting rRNAs while maintaining a similar transcriptome composition as Ribo-Zero across multiple species. Taken all together, we conclude that our DIY method provides a broadly applicable, customizable, and cost-effective technique for determining changes in bacterial gene expression patterns in a wide range of organisms and experimental contexts.

10.1128/mBio.00010-20.3 FIG S3 Correlation between counts per coding region before and after rRNA depletion for E. coli, B. subtilis, and C. crescentus total RNA. (A) Scatterplots showing correlation between read counts (RPKM) before and after rRNA depletion using our depletion strategy for E. coli (top left), B. subtilis (top right), and C. crescentus (bottom). All coding regions with at least 5 RPKM are shown (*n* = 767, 1,295, and 760). (B) Scatterplots comparing our depletion strategy and Ribo-Zero to undepleted RNA using a single RNA sample. Data plotted as in panel A (*n* = 498 for both plots). (C) Fold depletion for ratios of oligonucleotide to RNA and streptavidin bead/oligonucleotide ratios. Depletion was estimated by qRT-PCR of 16S and 23S, and an mRNA control gene relative to a bead-only negative control. Download FIG S3, TIF file, 2.1 MB. Copyright © 2020 Culviner et al. 2020 Culviner et al. This content is distributed under the terms of the Creative Commons Attribution 4.0 International license.

## DISCUSSION

We have developed a simple, fast, easy-to-implement, and cost-effective method for efficiently depleting rRNA from complex, total RNA samples. For three different species, E. coli, B. subtilis, and C. crescentus, we demonstrated robust depletion of 23S, 16S, and 5S rRNAs in a single step such that ∼70 to 90% of reads in RNA-seq arise from non-rRNA sources. This level of mRNA enrichment is sufficient for most RNA-seq studies. Our method showed relatively uniform depletion of rRNAs and minimal unwanted “off-targeting” of mRNAs compared to Ribo-Zero. Additionally, expression changes measured using our method correlated very strongly (*R*^2^ = 0.98 and 0.97; Fig. 3A and B) to those measured using the previously available Ribo-Zero kit. This strong correlation both validates our method and ensures that data generated via either method can be safely compared or combined.

Another method has recently been developed as an alternative to the discontinued Ribo-Zero kit (11). This method is based on hybridization of DNA oligonucleotides to rRNAs followed by digestion with RNase H, which recognizes DNA-RNA hybrids. This method also enabled robust rRNA depletion, although a direct comparison of RNA-seq counts per gene generated using this method and Ribo-Zero was not reported. Additionally, this alternative method requires extended (∼60-min) incubations with an RNase, albeit one that should be specific to DNA-RNA hybrids, whereas ours involves only hybridization and a precipitation step.

The set of biotinylated oligonucleotides tested here was designed to deplete the rRNA from a set of 8 selected organisms. These organisms span a large phylogenetic range so these oligonucleotides are likely broadly applicable to different bacterial species or even bacterial community samples. However, the set of oligonucleotides can also be easily optimized for a different species or set of species using the open-source software developed here and available on GitHub. In principle, inclusion of multiple oligonucleotides per RNA species to be depleted (11, 8, and 2 for the 23S, 16S, and 5S rRNAs, respectively) should enable depletion of even partially degraded rRNA, though here we did not test various levels of RNA degradation. If unwanted RNA species are observed in a final sample, additional oligonucleotides could be designed and added to deplete them. As noted, because the 5S rRNA is shorter and less conserved, probes specific to the 5S from a given species must typically be designed. However, the 5S rRNA does not yield nearly as many reads in RNA-seq data for a total RNA sample and may not require depletion for all studies.

In sum, the rRNA depletion methodology developed here should facilitate RNA-seq studies for any bacterium of interest. Notably, our method is also substantially less expensive than the Ribo-Zero kit. The cost of our method is ∼$10 per reaction to deplete 1 μg of total RNA (see Materials and Methods) compared to ∼$80 per reaction for Ribo-Zero. The cost for our approach stems primarily from the magnetic streptavidin-coated beads used to precipitate the biotinylated oligonucleotides bound to rRNA. Further optimization of the method reported here could likely reduce the cost further and possibly improve the extent of rRNA depletion. Nevertheless, as currently implemented, our method should enable the community to perform relatively easy, cost-effective, robust rRNA depletion, thereby facilitating RNA-seq studies.

## MATERIALS AND METHODS

### Oligonucleotide algorithm.

The algorithm was initialized with 500 and 1,000 oligonucleotides of 15 to 24 nucleotides in length for the 16S and 23S rRNA, respectively. Oligonucleotides were randomly positioned at nongapped locations of the alignment of the 8 species we selected. Sequences were chosen by randomly selecting a nucleotide matching one or more species at each position. Sequences were then optimized to achieve the target predicted *T_m_* of 62.5°C. *T_m_* calculations were conducted using the MeltingTemp module in the biopython library. We used the default nearest-neighbor calculation table for RNA-DNA hybrids (12). Notably, this model does not allow prediction of *T_m_* for some sequences with multiple sequential mismatches; as such, many oligonucleotides begin the optimization with undefined *T_m_*.

Optimization was conducted by sequential rounds of “mutation” on each oligonucleotide. Allowed mutations included moving the probe from 1 to 4 bases, shrinking the probe from 1 to 4 bases (on either end), extending the probe from 1 to 4 bases (on either end), or swapping the sequence of the oligonucleotide at one position to a nucleotide matching a different aligned rRNA. In each round of mutation, the starting oligonucleotide was mutated 25 times. From this set of mutated oligonucleotides, an oligonucleotide close to the target *T_m_* was chosen probabilistically (probabilities were determined by a normal distribution centered at 62.5°C with a standard deviation of 2°C). This probabilistic selection, coupled with the large number of oligonucleotides initialized, enables oligonucleotides to sample the possible binding locations without greedily descending on the first possible binding site they discover. Each oligonucleotide was mutated for 100 cycles before oligonucleotides binding to a number of sites across the 16S and 23S were selected.

To enable better binding of the more variable 23S 5′ and 3′ ends, we split the organisms into two groups (E. coli, P. aeruginosa, C. crescentus, and *R. parkeri*, and M. smegmatis, M. tuberculosis, B. subtilis, and S. aureus) and reran the optimization algorithm as described above. For each of these groups, we selected 2 additional oligonucleotides matching the 5′ and 3′ ends of the 23S.

Final oligonucleotides were hand-selected from those optimized by the algorithm. The general criteria we used to select oligonucleotides were as follows. Oligonucleotides should be spread across the sequence of the RNA to be depleted to allow depletion of partially degraded rRNA and maximize the number of binding sites. The *T_m_* of a given oligonucleotide should be >50°C for the majority of species to ensure that binding is maintained through the 50°C incubation step prior to elution. Where multiple oligonucleotides are possible in a single location (this is common as oligonucleotides tend to converge on conserved regions), relatively short oligonucleotides should be favored to reduce the possibility of off-target binding or oligonucleotide secondary structure. In some cases, designing 5S oligonucleotides to closely related species may be possible; in our hands 2 nonoverlapping 5S oligonucleotides meeting the above criteria and present at 2× the concentration of 16S and 23S oligonucleotides (a 4.5 rather than 2.25 ratio of probe to rRNA) effectively depleted 5S rRNA.

### Bacterial strains and culture condition.

E. coli MG1655 was grown to mid-log phase at 37°C in LB medium or M9 medium supplemented with 0.1% Casamino Acids, 0.4% glucose, 2 mM MgSO_4_, and 0.1 mM CaCl_2_. C. crescentus CB15N/NA1000 was grown to mid-log phase in peptone-yeast extract (PYE) medium at 30°C. B. subtilis 168 was grown to mid-log phase at 37°C in LB medium. For quantifying changes in expression from antibiotic treatment, cells were harvested 5 min after adding chloramphenicol or rifampicin at 50 μg/ml or 25 μg/ml, respectively.

### RNA extraction.

E. coli RNA was harvested by mixing 1 ml of cells with 110 μl of ice-cold stop solution (95% ethanol and 5% acid-buffered phenol) and spinning in a tabletop centrifuge for 30 s at 13,000 rpm. C. crescentus RNA was harvested by spinning down 2 ml of cells in a tabletop centrifuge for 30 s at 13,000 rpm. After removing the supernatant, pellets were flash-frozen and stored at −80°C until sample collection was complete. To extract RNA, TRIzol (Invitrogen) was heated to 65°C and added to each cell pellet. The mixtures were then shaken at 65°C for 10 min at 2,000 rpm in a thermomixer and flash-frozen at −80°C for at least 10 min. Pellets were thawed at room temperature and spun at top speed in a benchtop centrifuge at 4°C for 5 min. The supernatant was added to 400 μl of 100% ethanol and passed through a DirectZol spin column (Zymo). Columns were washed twice with RNA prewash buffer (Zymo) and once with RNA wash buffer (Zymo), and RNA was eluted in 90 μl diethyl pyrocarbonate (DEPC)-H_2_O. To remove genomic DNA, RNA was then treated with 4 μl of Turbo DNase I (Invitrogen) in 100 μl supplemented with Turbo DNase I buffer to a final concentration of 1X for 40 min at 37°C. RNA was then diluted with 100 μl DEPC H_2_O, extracted with 200 μl buffered acid phenol-chloroform, and ethanol precipitated at −80°C for 4 h with 20 μl of 3 M sodium acetate (NaOAc), 2 μl GlycoBlue (Invitrogen), and 600 μl ice-cold ethanol. Samples were centrifuged at 4°C for 30 min at 21,000 × *g* to pellet RNA and then washed twice with 500 μl of ice-cold 70% ethanol, followed by centrifugation at 4°C for 5 min. RNA pellets were then air dried and resuspended in DEPC-H_2_O. RNA yield was quantified by a NanoDrop spectrophotometer, and RNA integrity was verified by running 50 ng of total RNA on a Novex 6% Tris-buffered EDTA (TBE)–urea polyacrylamide gel (Invitrogen).

B. subtilis total RNA was harvested by mixing 5 ml of cell culture with 5 ml of cold (−30°C) methanol and spinning down at 5,000 rpm for 10 min. After removing the supernatant, pellets were frozen at −80°C. To lyse cells, pellets were vortexed in 100 μl lysozyme (10 mg/ml) in TE (10 mM Tris-HCl and 1 mM EDTA) at pH 8.0 and incubated for 5 min at 37°C. Lysates were cleared by adding 350 μl RLT buffer (Qiagen) in 1% beta-mercaptoethanol and vortexing. Lysates were then mixed with 250 μl ethanol, vortexed, and passed through an RNeasy mini-spin column (Qiagen). Columns were washed with 350 μl RW1 buffer (Qiagen). To remove genomic DNA, 40 μl of DNase I in RDD buffer (Qiagen) was applied to each column, and columns were incubated at room temperature for 15 min. Columns were then washed once with 350 μl RW1 buffer (Qiagen) and twice with RPE buffer (Qiagen), and RNA was eluted in 30 μl DEPC-H_2_O. RNA yield was quantified by a NanoDrop spectrophotometer, and RNA integrity was verified by running 50 ng of total RNA on a Novex 6% TBE-urea polyacrylamide gel (Invitrogen).

### rRNA depletion, DIY method.

Biotinylated oligonucleotides were selected using our algorithm, synthesized by IDT, and resuspended to 100 μM in TE buffer (Qiagen). An undiluted oligonucleotide mix for each organism was created by mixing equal volumes of all 16S and 23S primers, as well as double volumes of 5S primers. This undiluted mix was then diluted in DEPC-H_2_O based on the amount of total RNA added to the depletion reaction mixture, using an Excel-based calculator (available with code at https://github.com/peterculviner/ribodeplete).

Dynabeads MyOne streptavidin C1 beads (ThermoFisher) were washed three times in an equal volume of 1× B&W buffer (5 mM Tris HCl [pH 7.0], 5 mM Tris-HCl [pH 8.0], 500 μM EDTA, 1 M NaCl) and then resuspended in 30 μl of 2× B&W buffer (10 mM Tris-HCl [pH 7.0], 10 mM Tris-HCl [pH 8.0], 1 mM EDTA, 2 M NaCl). To prevent RNase contamination, 1 μl of SUPERase-In RNase inhibitor (ThermoFisher) was added to the beads. The beads were then incubated at room temperature until probe annealing (below) was complete.

To anneal biotinylated probes to rRNA, 2 to 3 μg total RNA and the diluted probe mix were combined on ice into an annealing reaction mixture containing 1× SSC (0.15 M NaCl plus 0.015 M sodium citrate) and 500 μM EDTA. The volume of RNA and probe mix added to each reaction mixture was determined using our Excel-based calculator. The mixtures were incubated in a thermocycler at 70°C for 5 min, followed by a slow ramp-down to 25°C at a rate of 1°C per 30 s. To pull down biotinylated probes bound to rRNA, annealing reaction mixtures were then added directly to beads in 2× B&W buffer, mixed by pipetting and vortexing at medium speed, and incubated for 5 min at room temperature. Reaction mixtures were then vortexed at medium speed, incubated at 50°C for 5 min, and then placed directly on a magnetic rack to separate beads from the remaining total RNA. The supernatant was pipetted away from the beads, placed on ice, and diluted to 200 μl in DEPC-H_2_O. RNA was then ethanol precipitated at −20°C for at least 1 h with 20 μl of 3 M NaOAc, 2 μl GlycoBlue (Invitrogen), and 600 μl ice-cold ethanol. Samples were centrifuged at 4°C for 30 min at 21,000 × *g* to pellet RNA and then washed twice with 500 μl of ice-cold 70% ethanol, followed by centrifugation at 4°C for 5 min. RNA pellets were then air dried and resuspended in 10 μl DEPC-H_2_O. RNA yield was quantified by a NanoDrop spectrophotometer, and the efficiency of rRNA depletion was verified by running 50 ng of total RNA on a Novex 6% TBE-urea polyacrylamide gel (Invitrogen).

### Optimization of rRNA depletion.

In the process of generating our depletion protocol, we tried multiple ratios of streptavidin-coated beads to biotinylated oligonucleotides and biotinylated oligonucleotides to total RNA (see Fig. S3C in the supplemental material). In brief, ratios were calculated assuming that 90% of total RNA by weight was rRNA. Thus, using the molecular weight of the E. coli ribosome and the micrograms of input RNA, the number of ribosomes can be estimated. The oligonucleotide/ribosome ratio is then the ratio of the number of moles of a single oligonucleotide in an equimolar mix of all oligonucleotides to the moles of ribosome in the input RNA. To calculate the oligonucleotide/streptavidin-coated bead ratio, the total moles of biotinylated oligonucleotides was compared to the theoretical binding capacity of the streptavidin beads (5 × 10^−12^ mol/μl). We found that rRNA was efficiently depleted across a range of ratios. However, it was critical to have a significant excess of streptavidin-coated beads over biotinylated oligonucleotides, as oligonucleotides that do not successfully capture rRNA also bind streptavidin more effectively, outcompeting bound rRNA-bound oligonucleotides and reducing rRNA capture efficiency. In addition, the presence of bound rRNA may interfere with proper bead binding. We selected our final ratios to achieve reliable depletion of rRNA at a low per-reaction cost. These ratios can be customized using the Excel-based calculator available on GitHub. Depletion of rRNA across multiple ratios were calculated using qRT-PCR of the 16S, 23S, and RplJ (as an mRNA control gene) (Fig. S3C) using primers listed in Table S1.

### Cost calculation.

The majority of reagents are common laboratory supplies for labs that work with RNA. To maintain the optimized ratio between streptavidin beads, biotinylated oligonucleotides, and rRNA, more oligonucleotides and beads must be used to deplete more total RNA. Considering the input, the cost per reaction is approximately $10, $19, or $28 for 1, 2, or 3 μg of RNA, respectively. The majority of the cost per reaction arises from streptavidin-coated magnetic beads; cost could likely be further decreased by using less-expensive streptavidin-coated beads or decreasing the quantity of beads used (see above). The upfront cost of purchasing oligonucleotides (IDT) is approximately $1,000 for large-scale synthesis or $500 for smaller-scale synthesis (available for sets of oligonucleotides >24). However, a single oligonucleotide synthesis order is adequate for hundreds of depletion reactions.

### RNA-seq library preparation.

Libraries were generated as described previously with a few modifications described below (13). The library generation protocol was a modified version of the paired-end strand-specific dUTP method using random hexamer priming. For libraries without rRNA removal, 500 ng of total RNA was used in the fragmentation step. For libraries with rRNA removal, 2 to 3 μg of input RNA was used in the rRNA removal step.

### rRNA depletion by Ribo-Zero.

rRNA depletion via Ribo-Zero treatment (Illumina) was conducted as described previously (13). Briefly, provided magnetic beads were prepared individually by adding 225 μl of beads to a 1.5-ml tube, left to stand on a magnetic rack for 1 min, washed twice with 225 μl of water, and resuspended in 65 μl of provided resuspension solution with 1 μl of provided RNase inhibitor. Samples were prepared using provided reagents with 4 μl of reaction buffer, 2 to 3 μg of total RNA, and 10 μl of rRNA removal solution in a total reaction volume of 40 μl. Samples were incubated at 68°C for 10 min and at room temperature for 5 min. Samples were added directly to the resuspended magnetic beads, mixed by pipetting, incubated for 5 min at room temperature, and then incubated for 5 min at 50°C. After incubation, samples were placed on a magnetic rack and the supernatant was transferred to a new tube, discarding the beads. Samples were ethanol precipitated as above with a 1-h incubation at −20°C and resuspended in 9 μl of water.

### Fragmentation.

RNA libraries were fragmented by adding 1 μl of 10× fragmentation buffer (Invitrogen) to 9 μl of input RNA in DEPC-H_2_O and heating at 70°C for 8 min. Fragmentation reactions were stopped by immediately placing reaction mixtures on ice and adding 1 μl of stop solution (Invitrogen). Reaction mixtures were diluted to 20 μl in DEPC-H_2_O, and RNA was ethanol precipitated at −20°C for at least 1 h with 2 μl of 3 M NaOAc, 2 μl GlycoBlue (Invitrogen), and 60 μl ice-cold ethanol. Samples were centrifuged at 4°C for 30 min at 21,000 × *g* to pellet RNA and then washed with 200 μl of ice-cold 70% ethanol, followed by centrifugation at 4°C for 5 min. RNA pellets were then air dried and resuspended in 6 μl DEPC-H_2_O.

### cDNA synthesis.

One microliter of random primers at 3 μg/μl (Invitrogen) was added to fragmented RNA, and the mixture was heated at 65°C for 5 min and placed on ice for 1 min. To conduct first-strand synthesis, 4 μl of first-strand synthesis buffer (Invitrogen), 2 μl of 100 mM dithiothreitol (DTT), 1 μl of 10 mM deoxynucleoside triphosphates (dNTPs), 1 μl of SUPERase-In (Invitrogen), and 4 μl of DEPC-H_2_O were added to each reaction mixture. Reaction mixtures were incubated at room temperature for 2 min, followed by addition of 1 μl of Superscript III. Reaction mixtures were then placed in a thermocycler for the following program: 25°C for 10 min, 50°C for 1 h, and 70°C for 15 min. To extract cDNA, reaction mixtures were diluted to 200 μl in DEPC-H_2_O and then vortexed with 200 μl of neutral phenol-chloroform-isoamyl alcohol. Following centrifugation, the aqueous layer was extracted, and cDNA was ethanol precipitated at −20°C for at least 1 h with 18.5 μl of 3 M NaOAc, 2 μl GlycoBlue (Invitrogen), and 600 μl ice-cold ethanol. Samples were centrifuged at 4°C for 30 min at 21,000 × *g* to pellet cDNA and then washed twice with 500 μl of ice-cold 70% ethanol, followed by centrifugation at 4°C for 5 min. Pellets were then air dried and resuspended in 104 μl DEPC-H_2_O. Second-strand synthesis was conducted by adding 30 μl of second-strand synthesis buffer (Invitrogen), 4 μl of 10 mM dNTPs (with dUTP instead of dTTP), 4 μl of first-strand synthesis buffer (Invitrogen), and 2 μl of 100 mM DTT to each sample, followed by incubation on ice for 5 min. To initiate second-strand synthesis, 1 μl of RNase H (NEB), 1 μl of E. coli DNA ligase (NEB), and 4 μl of E. coli DNA polymerase I (NEB) were added to each sample. Reaction mixtures were then incubated at 16°C for 2.5 h.

### End repair and adaptor ligation.

Cleanup for second-strand synthesis and all subsequent steps were conducted using Agencourt AMPure XP magnetic beads (Beckman Coulter), and beads were left in the reaction mixture to be reused for subsequent cleanup steps. For each sample, 100 μl of beads was added to 1.5-ml tubes and placed on a magnetic rack. The supernatant was removed and replaced with 450 μl of 20% (wt/vol) polyethylene glycol (PEG) 8000 in 2.5 M NaCl. Second-strand synthesis reaction mixtures were then added directly to resuspended beads, mixed by pipetting and vortexing, and incubated at room temperature for 5 min. Samples were then placed on a magnetic rack for ∼10 min, or until the solution was clear, and the supernatant was removed. Beads were then washed twice in 500 μl of 80% ethanol, dried, and resuspended in 50 μl of elution buffer (Qiagen). End-repair reactions were conducted by adding 10 μl of 10× T4 DNA ligase buffer (NEB), 4 μl of 10 mM dNTPs, 5 μl of T4 DNA polymerase (NEB), 1 μl of Klenow DNA polymerase (NEB), 5 μl of T4 polynucleotide kinase (NEB), and 25 μl of DEPC-H_2_O and incubating at 25°C for 30 min. To clean up the reaction mixtures, 300 μl of 20% (wt/vol) PEG 8000 in 2.5 M NaCl was mixed with each reaction mixture by pipetting and vortexing. Samples were then incubated at room temperature for 5 min and then placed on a magnetic rack for ∼5 min. The supernatant was removed, and the beads were then washed twice in 500 μl of 80% ethanol, dried, and resuspended in 32 μl of elution buffer (Qiagen). 3′-Adenylation reactions were conducted by adding 5 μl of NEB buffer 2 (NEB), 1 μl 10 mM dATP, 3 μl Klenow fragment (3′→5′ exo-) (NEB), and 9 μl of DEPC-H_2_O to each reaction mixture and incubating at 37°C for 30 min. To clean up the reaction mixtures, 150 μl of 20% (wt/vol) PEG 8000 in 2.5 M NaCl was mixed with each reaction mixture by pipetting and vortexing. Samples were then incubated at room temperature for 5 min and then placed on a magnetic rack for ∼5 min. The supernatant was removed, and the beads were then washed twice in 500 μl of 80% ethanol, dried, and resuspended in 20 μl of elution buffer (Qiagen). To elute DNA from the beads, reaction mixtures were incubated at room temperature for 5 min. Tubes were then returned to the magnetic rack and incubated for 1 to 2 min to allow the solution to clear, and then half of the supernatant (10 μl) was removed and stored at −20°C in case of downstream failure. To ligate adaptors to DNA, 1 μl of 5 μM annealed adaptors and 10 μl of Blunt/TA ligase master mix (NEB) were added to each reaction mixture, and reaction mixtures were incubated at 25°C for 20 min. Annealed adaptor mix was made by mixing 25 μl of a 200 μM solution of each paired-end adaptor together, heating to 90°C for 2 min, cooling at 2°C/minute for 30 min on a thermocycler, placing on ice, adding 50 μl of water, and storing aliquots at −20°C. To clean up ligation reaction mixtures, 60 μl of 20% (wt/vol) PEG 8000 in 2.5 M NaCl was mixed with each reaction mixture by pipetting and vortexing, and reaction mixtures were incubated at room temperature for 5 min. Reaction mixtures were then placed on a magnetic rack for ∼10 min, until solutions were clear, and the supernatant was removed. The beads were then washed twice in 500 μl of 80% ethanol, dried, and resuspended in 19 μl of 10 mM Tris-HCl (pH 8) and 0.1 mM EDTA. Reaction mixtures were then incubated at room temperature for 5 min to completely elute DNA. Tubes were then returned to the magnetic rack and incubated for 1 to 2 min to allow the solution to clear, then the supernatant was removed and moved to a new tube, and the beads were discarded. To digest the dUTP-containing second strand, 1 μl of USER enzyme (NEB) was added to 19 μl of eluted DNA and incubated at 37°C for 15 min, followed by heat inactivation at 95°C for 5 min.

### Library amplification.

PCR mixtures were prepared by mixing 10 μl of library template (diluted if too concentrated), 2 μl of 25 μM global primer, 2 μl of 25 μM barcoded primer, 11 μl of H_2_O, and 25 μl of 2× KAPA HiFi HotStart ReadyMix (Roche). Reaction mixtures were then cycled through the following thermocycler protocol: 98°C/45 s, 98°C/15 s, 60°C/30 s, 72°C/30 s, and 72°C/1 min. Steps 2 to 4 were repeated for 9 to 12 cycles, depending on the results of 10-μl optimization reaction mixtures. Following amplification, PCRs were run on an 8% TBE-polyacrylamide gel (Invitrogen) for 30 min at 180 V, and the region from 200 to 350 bp was excised, crushed, soaked in 500 μl 10 mM Tris (pH 8.0), and frozen at −20°C for at least 15 min. To elute DNA from the gel, reaction mixtures were shaken at 2,000 rpm for 10 min at 70°C in a thermomixer, followed by 1 h at 37°C. Reaction mixtures were then spun through a Spin-X 0.22-μm cellulose acetate column (Costar) and transferred to a new tube. Libraries were isopropanol precipitated by adding 32 μl 5 M NaCl, 2 μl GlycoBlue (Invitrogen), and 550 μl 100% isopropanol and incubating at −20°C for at least 1 h. Samples were then centrifuged at 4°C for 30 min at 21,000 × *g* to pellet DNA and then washed with 1 ml of ice-cold 70% ethanol, followed by centrifugation at 4°C for 5 min. DNA pellets were then air dried and resuspended in 11 μl H_2_O. Paired-end sequencing of amplified libraries was then performed on an Illumina NextSeq500, and single-end sequencing on an Illumina MiSeq.

### RNA-sequencing read mapping and normalization.

FASTQ files for each barcode were mapped to the E. coli MG1655 genome (NC_000913.2), the B. subtilis 168 genome (NC_000964.3), or the C. crescentus NA1000 genome (NC_011916.1) using bowtie2 (version 2.1.0) with the following arguments: -D 20 –R 3 –N 0 –L 20 –i S,1,0.50. The SAMtools (version 0.1.19) suite was used via the pysam library (version 0.9.1.4) for interconversion of BAM and SAM file formats and conducting indexing. Gene names and coding region positions were extracted from NCBI annotations.

### Single-end sequencing.

For all analyses except that of fragment density across E. coli rRNA loci (Fig. 2D), one count was added to the middle of each read. All reads mapping to a given coding region were then summed and normalized by reads per kilobase of transcript per million (RPKM). This normalized quantity was then used in all downstream analyses.

For analysis of fragment density across rRNA loci, one count was added for all positions between and including the 5′ and 3′ ends of reads. To correct for variability in sequencing depth, counts at each position were divided by a sample size factor. Briefly, counts recorded in each genomic region were summed for all samples and then the geometric mean was taken across samples to yield a reference sample. The size factor for a given sample was the median counts in all regions after normalizing counts to the reference samples.

### Analysis of oligonucleotide depletion efficiency.

To quantify the efficiency of rRNA depletion, the sum of reads mapping to rRNA loci was divided by the total number of mapped reads in each sample. To compare the reads mapping to individual coding regions following rRNA depletion and/or antibiotic treatment (Fig. 3A to C; see also Fig. S2B to D and Fig. S3A and B in the supplemental material), coding regions were filtered for expression by RPKM, and then the correlations between RPKM for individual coding regions were compared using the SciPy statistical functions package. Outliers for the ratio of reads per coding region following Ribo-Zero versus DIY treatment (Fig. S2B) were identified by measuring the distance for all genes in Cartesian coordinates from the log-log least-squares fit for all regions above the expression threshold. Outliers were defined as genes for which this ratio was less than or greater than 2 standard deviations from the mean line. Outliers in Fig. 3C and Fig. S2D were hand-picked.

### Data and code availability.

The code used to generate the oligonucleotides is available for download at https://github.com/peterculviner/ribodeplete. The raw and processed sequencing data are available on GEO (GSE142656).

10.1128/mBio.00010-20.5 TABLE S2 mRNA outliers following rRNA depletion. Download Table S2, XLSX file, 0.02 MB. Copyright © 2020 Culviner et al. 2020 Culviner et al. This content is distributed under the terms of the Creative Commons Attribution 4.0 International license.

## References

[1] Hör J, Gorski SA, Vogel J 2018 Bacterial RNA biology on a genome scale. Mol Cell 70:785–799. doi:10.1016/j.molcel.2017.12.023. 29358079

[2] Croucher NJ, Thomson NR 2010 Studying bacterial transcriptomes using RNA-seq. Curr Opin Microbiol 13:619–624. doi:10.1016/j.mib.2010.09.009. 20888288 PMC3025319

[3] Creecy JP, Conway T 2015 Quantitative bacterial transcriptomics with RNA-seq. Curr Opin Microbiol 23:133–140. doi:10.1016/j.mib.2014.11.011. 25483350 PMC4323862

[4] Podnar J, Deiderick H, Huerta G, Hunicke-Smith S 2014 Next-generation sequencing RNA-Seq library construction. Curr Protoc Mol Biol 106:4.21.1–4.21.19. doi:10.1002/0471142727.mb0421s106. 24733242

[5] Nagalakshmi U, Wang Z, Waern K, Shou C, Raha D, Gerstein M, Snyder M 2008 The transcriptional landscape of the yeast genome defined by RNA sequencing. Science 320:1344–1349. doi:10.1126/science.1158441. 18451266 PMC2951732

[6] Mortazavi A, Williams BA, McCue K, Schaeffer L, Wold B 2008 Mapping and quantifying mammalian transcriptomes by RNA-Seq. Nat Methods 5:621–628. doi:10.1038/nmeth.1226. 18516045 PMC13303166

[7] Westermann AJ, Gorski SA, Vogel J 2012 Dual RNA-seq of pathogen and host. Nat Rev Microbiol 10:618–630. doi:10.1038/nrmicro2852. 22890146

[8] Herbert ZT, Kershner JP, Butty VL, Thimmapuram J, Choudhari S, Alekseyev YO, Fan J, Podnar JW, Wilcox E, Gipson J, Gillaspy A, Jepsen K, BonDurant SS, Morris K, Berkeley M, LeClerc A, Simpson SD, Sommerville G, Grimmett L, Adams M, Levine SS 2018 Cross-site comparison of ribosomal depletion kits for Illumina RNAseq library construction. BMC Genomics 19:199. doi:10.1186/s12864-018-4585-1. 29703133 PMC6389247

[9] Stewart FJ, Ottesen EA, Delong EF 2010 Development and quantitative analyses of a universal rRNA-subtraction protocol for microbial metatranscriptomics. ISME J 4:896–907. doi:10.1038/ismej.2010.18. 20220791

[10] He S, Wurtzel O, Singh K, Froula JL, Yilmaz S, Tringe SG, Wang Z, Chen F, Lindquist EA, Sorek R, Hugenholtz P 2010 Validation of two ribosomal RNA removal methods for microbial metatranscriptomics. Nat Methods 7:807–812. doi:10.1038/nmeth.1507. 20852648

[11] Huang Y, Sheth RU, Kaufman A, Wang HH 2020 Scalable and cost-effective ribonuclease-based rRNA depletion for transcriptomics. Nucleic Acids Res 48:e20. doi:10.1093/nar/gkz1169. 31879761 PMC7038938

[12] Sugimoto N, Nakano S, Katoh M, Matsumura A, Nakamuta H, Ohmichi T, Yoneyama M, Sasaki M 1995 Thermodynamic parameters to predict stability of RNA/DNA hybrid duplexes. Biochemistry 34:11211–11216. doi:10.1021/bi00035a029. 7545436

[13] Culviner PH, Laub MT 2018 Global analysis of the E. coli toxin MazF reveals widespread cleavage of mRNA and the inhibition of rRNA maturation and ribosome biogenesis. Mol Cell 70:868–880.e10. doi:10.1016/j.molcel.2018.04.026. 29861158 PMC8317213

