# Supplementary figures and images for: A Simple, Cost-Effective, and Robust Method for rRNA Depletion in RNA-Sequencing Studies
Source: mBio. 2020 Apr 21;11(2):e00010-20. doi: 10.1128/mBio.00010-20 (PMC7175087; doi:10.1128/mBio.00010-20)

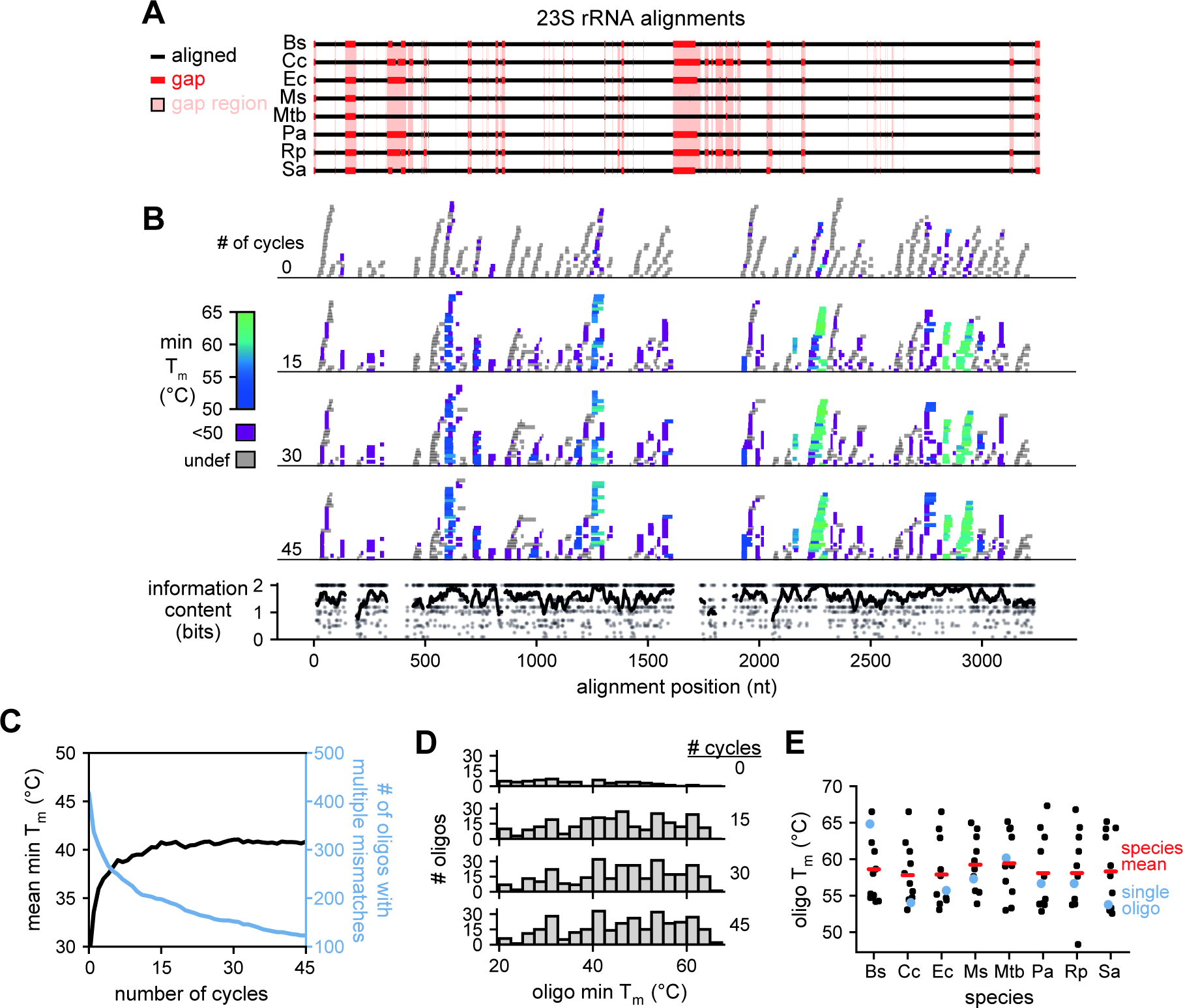

Supplement: FIG S1 [file mBio.00010-20-sf001.tif]

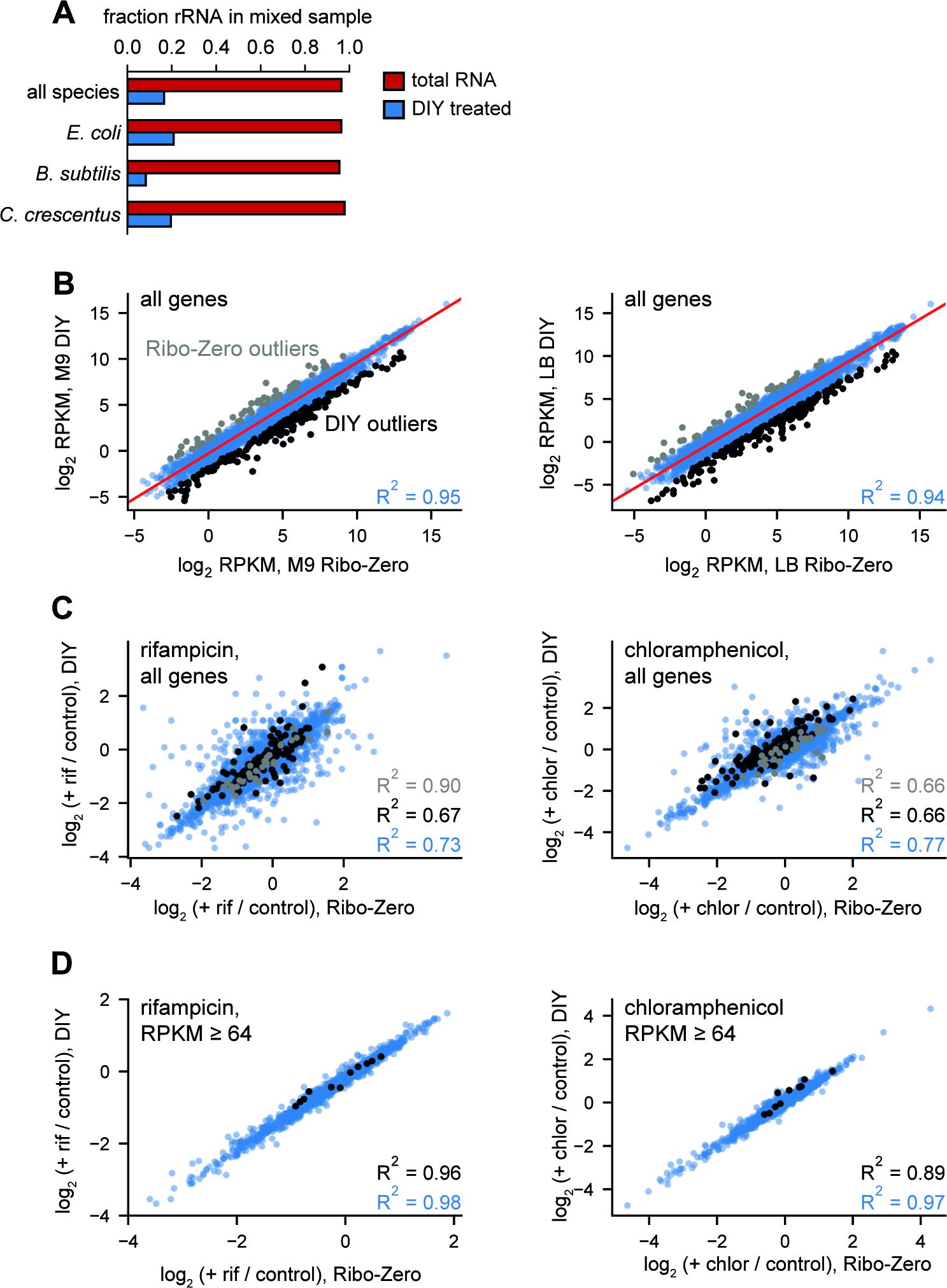

Supplement: FIG S2 [file mBio.00010-20-sf002.tif]

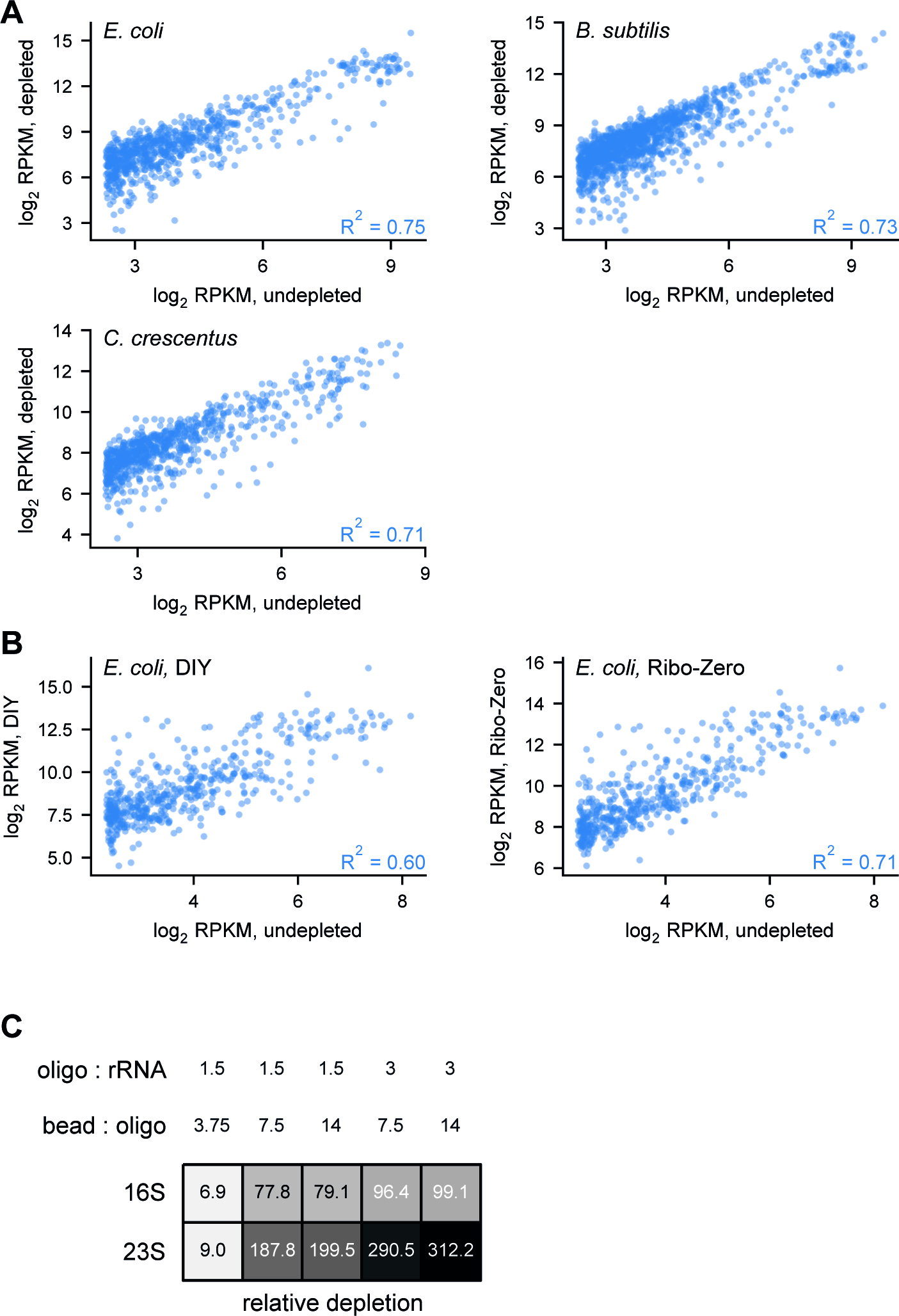

Supplement: FIG S3 [file mBio.00010-20-sf003.tif]
