# Supplementary material for: A Simple, Cost-Effective, and Robust Method for rRNA Depletion in RNA-Sequencing Studies
Source: mBio. 2020 Apr 21;11(2):e00010-20. doi: 10.1128/mBio.00010-20 (PMC7175087; doi:10.1128/mBio.00010-20)
Supplement: TABLE S1 [file mBio.00010-20-st001.pdf]

Table S1 - Sequences of oligonucleotides for bacterial rRNA depletion and qRT-PCR

Name	Sequence
23S_1	ACCTTTCCCTCACGGTACTGGTTCGCTATCGGTCA
23S_2	AGTCGCTGGCTCATTATACAAAAGGTACGCCGTCACC
23S_3	TCGGGGAGAACCAGCTATCTCCGGGTTTGATTGGC
23S_4	GTGGCTGCTTCTAAGCCAACATCCTG
23S_5	GGGTACAGGAATATTAACCTGATTTCCATCGACTACGCC
23S_6	CACCTGTGTCGGTTTGGGGTACGGT
23S_7	TCGTGCGGGTCGGAACTTACCCGACAAG
23S_8	GAGCCGACATCGAGGTGCCAAACA
23S_9	CGGCGGATAGGGACCGAACTGTCTCACGAC
16S_1	CCGCTCGACTTGCATGTGTTAAGCATGCCGACAGCGTTCG
16S_2	CCCATTGTGCAAGATTCCCTACTGCTGCCTCCCGT
16S_3	ACCGCGGCTGCTGGCACGGAGT
16S_4	ACGGCGTGGACTACCAGGGTAT
16S_5	TCCACATGCTCCACCGCTTGTGCGGGCCCCCG
16S_6	ACCCAACATCTCACAACACGAGCTGACGACA
16S_7	GGGCAGTGTGTACAAGGCCCGGGA
16S_8	AAGGAGGTGATCCAGCCGCAG
23S_GN1	CACGTCCTTCATCGCCTTTTACTGCCAAGGCATCC
23S_GN2	CCACACCCGGCCTATCAACGTGGTGGTCTTCGACG
23S_GP1	ATGCCAAGGCATCCACCATGCGCCCT
23S_GP2	TATCCTGTCCGCACGTGGCTACCCAGCG
5S_EcPa_1	GTTCGGGAAGGGGTCAGGTGGGTCCAACGCGCTA
5S_EcPa_2	AGACCCCACACTACCATCGGCGATACGTCG
5S_Sa_1	GCATGGGAACAGGTGTGACCTCCTTGCTAT
5S_Sa_2	GCGGAACGTAAGTTCGACTACCATCGACGCT
5S_Bs_1	GGTATGGGAACGGGTGTGACCTCTTCGCTA
5S_Bs_2	CGACTACCATCGGCGCTGAAGAGCTTAAC
5S_Cc_1	CCGAGTTCGGAATGGGATCGGGTGGG
5S_Cc_2	CTTGAGACGAAGTACCATTGGCCCAGGG
5S_Rp_1	GGATGGGATCGTGTGTTTCACTCATGCTATAACCACC
5S_Rp_2	TCCCATGCCTTATGACATAGTACCATTAGCGCTAT
5S_MtbMs_1	ACCGGGCGTTTCCCTGCCGCTA
5S_MtbMs_2	GGTAGTATCATCGGCGCTGGCAGG
rplJ_PCR_F	TCGCGAAGCTGGCGTAT
rplJ_PCR_R	ATCAGGGTCGGACCAACAAA
16S_PCR_F	CCATGGGAGTGGGTTGC
16S_PCR_R	CAGTCATGAATCACAAAGTGGTAAG
23S_PCR_F	ATCACTGGTGTTCGGGTTGT
23S_PCR_R	TCAACGTCGTCGTCTTCAAC
